# Personalized tissue-engineered arteries as vascular graft transplants: A safety study in sheep

**DOI:** 10.1016/j.reth.2022.08.005

**Published:** 2022-09-07

**Authors:** Lachmi Jenndahl, Klas Österberg, Yalda Bogestål, Robin Simsa, Tobias Gustafsson-Hedberg, Patrik Stenlund, Sarunas Petronis, Annika Krona, Per Fogelstrand, Raimund Strehl, Joakim Håkansson

**Affiliations:** a VERIGRAFT AB, Arvid Wallgrensbacke 20, 413 46, Göteborg, Sweden; b Sahlgrenska Academy, Institution of Medicine, Department of Molecular and Clinical Medicine, Blå Stråket 5 B Wallenberg Laboratory, 41345 Gothenburg, Sweden; c RISE Research Institutes of Sweden, Materials and Production, Brinellgatan 4, 504 62 Borås, Sweden; d Department of Molecular and Clinical Medicine/Wallenberg Laboratory, University of Gothenburg and Sahlgrenska University Hospital, Gothenburg, Sweden; e RISE Research Institutes of Sweden, Agriculture and Food, Box 5401, 402 29 Gothenburg, Sweden; f Gothenburg University, Department of Laboratory Medicine, Institute of Biomedicine, Gothenburg, Sweden

**Keywords:** ATMP, Blood vessels, Recellularization, Regenerative medicine, Scaffold, Tissue engineering

## Abstract

Patients with cardiovascular disease often need replacement or bypass of a diseased blood vessel. With disadvantages of both autologous blood vessels and synthetic grafts, tissue engineering is emerging as a promising alternative of advanced therapy medicinal products for individualized blood vessels. By reconditioning of a decellularized blood vessel with the recipient’s own peripheral blood, we have been able to prevent rejection without using immunosuppressants and prime grafts for efficient recellularization *in vivo.* Recently, decellularized veins reconditioned with autologous peripheral blood were shown to be safe and functional in a porcine *in vivo* study as a potential alternative for vein grafting. In this study, personalized tissue engineered arteries (P-TEA) were developed using the same methodology and evaluated for safety in a sheep *in vivo* model of carotid artery transplantation. Five personalized arteries were transplanted to carotid arteries and analyzed for safety and patency as well as with histology after four months *in vivo.* All grafts were fully patent without any occlusion or stenosis. The tissue was well cellularized with a continuous endothelial cell layer covering the luminal surface, revascularized adventitia with capillaries and no sign of rejection or infection. In summary, the results indicate that P-TEA is safe to use and has potential as clinical grafts.

## Highlights

• Safety and functionality evaluation of a tissue engineered ATMP in a sheep model of carotid transplantation.
• Efficient cellularization of a personalized tissue engineered artery *in vivo.*
• Personalized tissue engineered artery fully patent after four months *in vivo.*

## 1 Introduction

For patients in need of cardiac or peripheral vascular bypass grafting, autologous blood vessels are today the primary choice. However, graft harvesting is associated with prolonged operation time and increased risk of wound complications due to surgical preparation. In the majority of coronary artery bypass grafting (CABG) procedures, vein grafts are harvested from the lower limb which for many patients result in surgical site infections (SSI), nerve injuries or persistent leg swelling. Also, many patients do not have suitable vessels and autograft suitability may be difficult to define in advance of the surgical procedure. In bypass surgery for peripheral arterial disease (PAD), autologous vein grafts are not only associated with prolonged procedural time and increased wound complications, but also a significant rate of technical failure related to venous valve destruction and side branch ligation [4]. Such adverse vascular events lead to a significant decrease in the patients’ quality of life and severe graft failure can lead to major amputation. There is a large need of suitable grafts for bypass surgeries with small diameter grafts (≤6 mm) since current synthetic grafts on the market suffer from patency problems and infections [10,27,41,42]. An ideal artificial vascular graft should be easy to surgically insert, biocompatible, non-thrombogenic, and the biomechanical properties should meet the demands of arterial blood pressure. For practical reasons, an off-the-shelf product is also preferable to avoid long waiting time before surgery for the patient. As an alternative to autologous and synthetic grafts, tissue engineering has emerged and shown promising progress over the last two decades. One possible method is decellularization of native blood vessels which can be applied on donated human or animal blood vessels by physical force, detergents, enzymes or other methods [9]. The ECM has reduced immunogenic properties compared with cellular antigens and is believed to be better accepted by the recipient's immune system [44]. Decellularized grafts have been evaluated as an option for vascular transplants, but many studies experience problems with graft-related thrombosis, infection, and aneurysm [22,32]. Establishment of an endothelial cell layer on the luminal surface of the vascular graft is considered crucial, as their release of several factors prevent coagulation [3] and the coverage of the extracellular matrix inhibit formation of intima hyperplasia [17].

Personalization of vascular grafts has potential to provide a reproducible method for improved *in vivo* outcome. Perfusion of functional organs with whole blood or blood-derived solutions preceding transplantation has been shown favorable for maintaining organ physiology and functionality [12,39]. We have previously shown that reconditioned tissue engineered veins, perfused with the recipient’s own peripheral blood after decellularization, were safe and functional with efficient recellularization *in vivo* and full patency in a pig model of *vena cava* transplantation over one month [14]. The rationale for this method is that autologous blood components from the recipient can attach to the surface of the decellularized scaffold to mask exposed extracellular matrix to make the graft more autologous and thereby prime for exposure to the blood stream and prevent rejection and thrombotic events and facilitate rapid cellularization *in vivo* after transplantation. The promising results on vein grafts motivated us to evaluate the same methodology on small diameter arterial grafts. VERIGRAFT is developing clinical-grade grafts under the trade name personalized tissue-engineered arteries (P-TEA) and we have used the P-TEA protocols for decellularization and reconditioning to produce sheep P-TEA. The grafts were evaluated for safety in a sheep model of carotid artery transplantation over four months. All grafts were well cellularized, tolerated by the host and fully patent without any occlusion or stenosis.

## 2 Materials and methods

### 2.1 Preparation of P-TEA

Artery segments were decellularized as previously described [36,37]. Briefly, carotid arteries were collected from sheep slaughter waste, stored in PBS containing 0.5× antibiotic-antimycotic (AA, Thermo Fisher Scientific) and frozen at −80 °C until further use. The arteries were thawed, connected to a perfusion bioreactor system and perfused with reagents at 37 °C under agitation of 115 rpm and perfusion speed of 100 mL/min. The artery segments were perfused for 24 h with 1% Triton X (Merck Millipore), followed by 8 h in 1% Tri-n-butyl phosphate (TnBP, Merck Millipore) and 16 h in 40 U/mL DNase (Thermo Fisher Scientific). Between each step, the blood vessel was washed in H_2_O and the whole procedure was repeated once. After two cycles, the arteries were washed in 5 mM EDTA (Merck Millipore) for 48 h followed by PBS for 24 h. The decellularized arteries were removed from the bioreactor, biopsies were taken for DNA quantification, histology and immunohistochemistry before peracetic acid sterilization and final washes in PBS under sterile conditions. Sterilized vessels were frozen at −80 °C or used directly for reconditioning.

Reconditioning of artery scaffolds were performed as previously described [14]. Briefly, decellularized arteries were connected to a reconditioning bioreactor and perfused with PBS + AA for 24 and 1 h with 50 IU/mL heparin (Leo Pharma) in PBS + 0,5 × AA. 25 mL peripheral blood was collected from respective sheep in heparin vacutainers (BD) and mixed with 25 mL STEEN Solution (XVIVO Perfusion), 0.5 × AA (Thermo Fisher Scientific), 80 ng/mL vascular endothelial growth factor (Cellgenics) and 10 ng/mL fibroblast growth factor (R&D Systems). 5 μg/mL acetylsalicylic acid (Sigma-Aldrich) was added to terminally inhibit all contained thrombocytes. The complete blood solution was added to the decellularized blood vessel, which allowed circulation of the blood solution through and around the vessel in a vertical position at 2 mL/min. This process was performed in a laminar flow hood at room temperature for seven days, during which the glucose level was measured with a Contour XT glucose meter (Bayer) and adjusted up to 8 mM with glucose solution (Life technologies, USA) when needed. After perfusion, the reconditioned artery (now called P-TEA) was harvested, rinsed with PBS, biopsied for DNA quantification and histology and kept in PBS + AA on ice until use (maximum 2 h). During blood procurement and production, a system was used to ensure traceability of the autologous sheep blood and the resulting graft to allow administration in an individualized, strictly autologous manner.

### 2.2 Sheep *in vivo* model for carotid artery transplantation of P-TEA

**Fig. 1:**
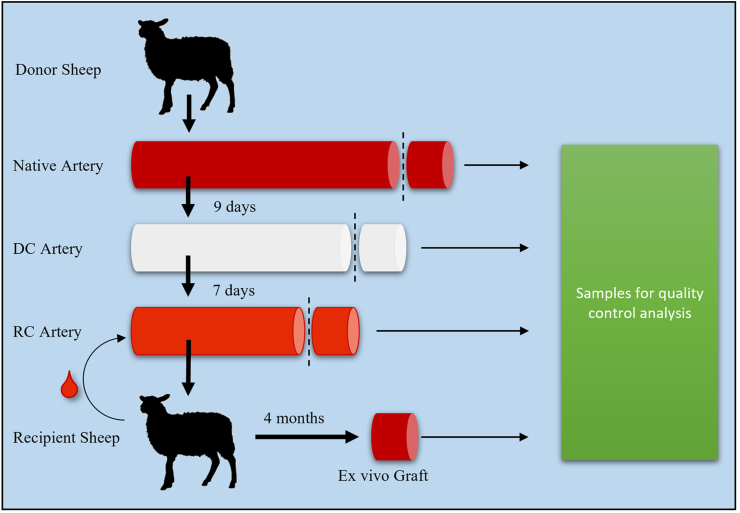
**Schematic overview of the graft through experimental process.** Carotid arteries are donated from sheep slaughter waste, decellularized over 9 days, reconditioned with the recipient sheep’s peripheral blood over seven days and the personalized tissue engineered artery is transplanted to the carotid artery. Four months after surgery, the graft is excised. Samples for quality control are harvested in all steps. DC = Decellularization, RC = Reconditioning.

The *in vivo* experiments were performed after prior approval from the local ethics committee for animal studies at the administrative court of appeals in Gothenburg, Sweden. Seven female sheep (bodyweight 59–73 kg) were used in this study; two for sham surgery (the sheep’s own *arteria carotis* was cut out and re-sutured with end-to-end anastomosis) and five P-TEA transplantations. The sheep were cared for in accordance with regulations for the protection of laboratory animals and were housed together before and after surgery. Clopidogrel (Plavix, Sanofi) 75 mg was given orally and Dalteparin (Fragmin, Pfizer) 12,500 IU up to 65 kg and 15,000 IU above 65 kg was given with subcutaneous injection. The medication was given once daily in the morning during the whole study starting two days before surgery. The only exception was that Dalteparin was given in the afternoon/evening the day of surgery. All surgical procedures were carried out under sterile conditions and under isoflurane anesthesia. Anesthesia was induced with injection of Dexmedetomidine 15 μg/kg (Dexdomitor, Orion Pharma Animal Health), and Propofol 20–40 mL (10 mg/mL) (Promea vet, Orion Pharma Animal Health). The sheep was then intubated and given inhalation anesthesia with isoflurane (Attane vet, VM Pharma). For pain relief, Buprenorphine 30 μg/kg (Vetergesic vet, Orion Pharma Animal Health) and Carprofen 4 mg/kg (Norocarp vet, N-vet) was given administered intramuscularly. Amoxicillin 15 mg/kg (Vetrimoxin vet, Cava Animal Health) was given as intramuscular injection as antibiotic prophylaxis. An incision was made on the left side of trachea and *arteria carotis* was dissected free from surrounding tissue. At the time of implantation, heparin 5000 IU (Leo Pharma) was administered intra venously before clamping of the artery. Five to seven cm segments (stretched to approximately nine cm after implantation) P-TEA were implanted by end-to-end anastomosis to the native carotid using 6-0 Prolene (Ethicon) single sutures. In the sham-operated sheep, the procedure was performed in the same way, but after dissected free, the native artery was cut twice at a distance of 5 cm and then sutured back. Metal clips were sutured outside of the blood vessel at the anastomosis sites for facilitated orientation under x-ray imaging. The soft tissue was closed with 3-0 Vicryl (Ethicon) and the skin was closed with a 3-0 Monocryl (Ethicon) suture intradermally. Ultrasound analysis of the P-TEA patency was performed on the sheep two weeks and two months post-surgery as well as in conjunction with euthanization four months post-surgery. At euthanization, angiography was performed under isoflurane anesthesia. The contralateral carotid artery was cannulated, and contrast was injected through the artery into the aortic arch for subsequent filling of both carotid arteries making both carotid arteries visible on the angiogram. Native samples, and the operated segment of *arteria carotis*, were excised and prepared for further analysis. The process from donated artery to excision of the transplanted P-TEA four month after surgery is schematically illustrated in Fig. 1.

### 2.3 DNA quantification

DNA quantification was performed by DNA extraction from 10 to 25 mg wet tissue samples from native, decellularized and reconditioned arteries, utilizing the DNeasy Blood&Tissue kit (Qiagen). Extracted DNA was quantified with Qubit Fluorometer and Qubit dsDNA HS assay kit (Life technologies), according to the manufacturer’s instructions.

### 2.4 Histology and immunohistochemistry

Samples of native, decellularized, reconditioned and explanted P-TEAs were fixated in 4% Formaldehyde for 24–72 h, embedded in paraffin and sectioned with 5 μm thickness. Samples were rehydrated following standard procedures and stained with 25 μg/mL DAPI (4′,6-diamidino-2-phenylindole) or hematoxylin-eosin (H&E) to observe the presence of nuclei in the tissue. As a complement to H&E staining, paraffin sections were also stained with Verhoeff-van Gieson, to visualize elastin fibers, and von Kossa staining to observe potential calcium deposits. 1Images were taken with a fluorescence microscope (Zeiss Axiovert 40 CFL). Immunohistochemical stainings were performed on paraffin sections by rehydrating samples following standard procedures and performing antigen retrieval by incubating samples for 15 min in a 95 °C water bath in a Tris–EDTA buffer (10 mM Tris Base, 1 mM EDTA Solution, 0.05% Tween 20, pH 9.0). The sections were blocked with 10% FBS (Gibco) for 30 min at room temperature and incubated with primary antibodies against CD31 (1:25, mouse, ab28364, Abcam), CD45 (1:50, rabbit, MCA2220GA, Biorad) and alpha smooth muscle actin (ASMA, 1:100, rabbit, ab7817, Abcam) over night at 4 °C in a moist chamber. Samples were rinsed 3 × 5min in PBS and incubated for 1 h with secondary anti-mouse (1:200, A21202, ThermoFisher) or anti-rabbit (1:200, A21207, ThermoFisher) antibodies conjugated with Alexa Fluor 488 or 594.

Then, slides were mounted with a drop of prolong antifade (ThermoFisher, USA) and visualized with a fluorescent microscope.

### 2.5 Confocal laser scanning microscopy

Sections were de-paraffinized at 60 °C in oven until the paraffin was melted. The sections were rehydrated in xylene two times for 10 min and incubated for 5 min each as follows: 99% ethanol, 99% ethanol, 96% ethanol, 70% ethanol and ddH2O. Antigen retrieval was performed by heating the samples in Tris EDTA, pH 9, 0,5% Tween at 98 °C for 15min. The sections were blocked with 5% BSA (Sigma-Aldrich) in PBS for 30 min followed by incubation overnight at 4 °C with primary antibody solution. Primary antibodies against aSMA (ab7817 mouse) and CD31 (ab28364 rabbit) were diluted 1:25 and 1:50, respectively, in PBS with 0.5% BSA, either one by one (single-labelling) or mixed together (double-labelling). Samples were rinsed 3 × 5 min in PBS and incubated for 2 h with secondary antibodies conjugated with Alexa Fluor Plus 488 goat anti-mouse IgG (H + L) (Invitrogen) and Alexa Fluor 546 Goat anti-Rabbit IgG (H + L) (Invitrogen) for aSMA and CD31 respectively, diluted 1:100 in PBS with 0.5% BSA (Sigma-Aldrich). The sections were rinsed 3 × 5 min in PBS and stained with SYTOX™ Deep Red Nucleic Acid Stain (Invitrogen), washed in PBS and mounted with ProLong DiamondTM Antifade Mountant (Invitrogen). The sections were examined in a confocal laser scanning microscope (SP5, TSC Leica) using a HCX PL APO lambda blue 20.0 × 0.70 IMM UV objective with zoom 1× and 4×. Excitation was performed with a 488 nm argon laser, a 543 nm HeNe laser and a 633 nm HeNe laser.

### 2.6 Biomechanical testing

Biomechanical properties of blood vessels were determined with a ring tensile test. Measurements of native, decellularized, sterilized, reconditioned and excised grafts four months after transplantation were performed on four different arteries. Samples were taken within a single vessel to avoid biological variability between donors. The same measurements were also performed on two sham operated arteries. Each artery was divided in three sections (A–C) for which three ring segments were cut off and selected for testing after each procedure (Supplementary Fig. 1). In addition, implanted P-TEA were excised four months after transplantation, cut into equally sized ringlets and analyzed directly after explanation. All ring segments were cut using a 3 mm guide template and the resulting length was measured by a digital caliper. All ringlets were subjected to ring tensile testing (Planar Biaxial TestBench Instrument, TA Instruments – ElectroForce System Group) in PBS solution, conducted at room temperature. The individual ringlets were mounted on two metal pins, each 2 mm in diameter, through the lumen of the artery where one pin was stationary while the other was displaced at constant speed until failure. The samples were preloaded to 0.03 N and allowed to adapt for 30 s. The initial sample internal diameter, later used in strain computation, was defined at the preload state. The construct was tested to failure at a speed of 40 mm/min whilst time and displacement were recorded, and load was measured either by a 22.2 or a 222 N load cell depending on the sample characteristics using the WinTest 7.01 software (TA Instruments – ElectroForce System Group). The internal pressure P was calculated using Laplace’s law which basic statement is given in Eq. (1) and the engineering stress given in Eq. (2). In these equations, T is the wall tension by unit length, σ_θ_ is the wall circumferential stress, t is the wall thickness, R_i_ and D_i_ the internal radius and diameter respectively, F is the measured load and L_0_ is the initial length of the ring segment.

(1) $$T=\sigma_{\theta }t=PR_{i}$$

(2) $$\sigma_{\theta }=\frac{F}{2L_{0}t}$$

(3) $$P=\frac{F}{L_{0}D_{i}}$$

The sample diameter changes with the displacement as the analysis progresses and since the latter is accurately known, it can be used in Eq. (3) to provide a better estimate of the actual pressure. In the case of burst pressure, the load at failure, F_max_, was used in Eq. (3). Additionally, the initial diameter, D_i_, was redefined as the diameter at failure, D_f,_ since it has been shown appropriate when estimating burst pressure by the ring tensile test method [18]. The burst pressure, the failure strain, and the stiffness of the construct were calculated from the biomechanical testing.

### 2.7 Scanning electron microscopy

Tissue samples were rinsed in PBS and submerged in 2.5% glutaraldehyde fixative (Sigma-Aldrich) at room temperature. After 2 h, the samples were placed in a fresh fixative of the same concentration and left in a fridge at 5 °C for 24 h. The samples were washed with buffer and submerged in 1% osmium tetroxide (Sigma-Aldrich) for 24 h at room temperature for secondary fixation. The samples were rinsed in deionized water, plunge-frozen in liquid propane and freeze-dried overnight in VirTis Sentry 2.0 Benchtop Freeze Dryer (SP Scientific). Before the SEM analysis, samples were coated with a 15 nm thin Au/Pd film using Gatan Model 682 PECS sputter-coater to prevent the charging under electron beam. The imaging was performed with a Zeiss Supra 40VP SEM, in secondary electrode image mode. The acceleration voltage and working distance were 4.03 kV and 9–12 mm, respectively.

### 2.8 Statistical analysis

Statistical analysis was performed with the SPSS software. For comparison of multiple groups, ANOVA test with Turkey HSD Post hoc for multiple comparison was used.

## 3 Results

### 3.1 Decellularization and reconditioning of vascular grafts

One artery was prepared for each of the five transplantations, and all processes were successfully performed. The length of the grafts before implantation were 50–70 mm and luminal diameter was 3.3–3.9 mm (Supplementary Table 1 and Supplementary Fig. 2). Measurements of DNA content in tissue from native sheep carotid artery, decellularized vessels and after reconditioning with the recipient sheep’s own blood revealed that decellularization reduced the DNA content from 85.2 ± 7.7 ng DNA/mg tissue in the native tissue to 0.3 ± 0.1 ng DNA/mg tissue in the decellularized artery (Supplementary Fig. 3). Further, reconditioning statistically significantly increased the DNA content to 9.8 ± 2.2 ng DNA/mg tissue.

### 3.2 Surgical transplantation of arterial graft and patency after four months *in vivo*

**Fig. 2:**
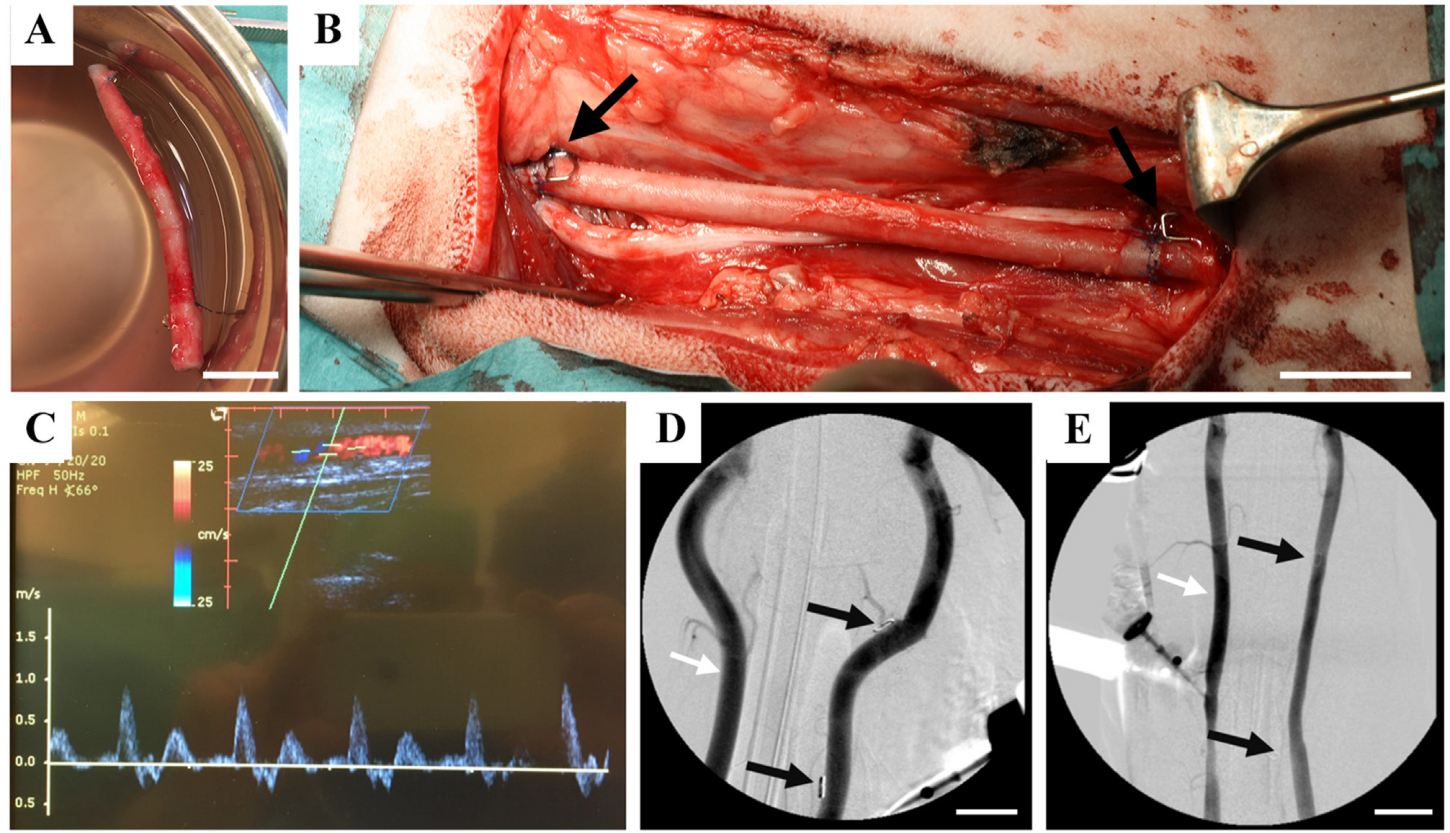
**Transplantation of carotid artery on sheep and patent blood vessel four months after transplantation.** (A) Reconditioned personalized tissue engineered artery (P-TEA) before transplantation. (B) Transplantation of the P-TEA to the carotid artery of sheep with end-to-end anastomosis. (C) Representative ultrasound image of P-TEA four months after surgery showing the patent artery with the graph illustrating adequate blood flow. (D, E) shows angiography from sham and P-TEV, respectively, with patent blood vessels without stenosis or occlusion. Black arrows in B, D and E marks metal clips sutured on the outside of the anastomosis for localization of the surgical site during angiography. Left vessel in D and E (marked with white arrow), shows the native non-operated artery at the other side of trachea. Scale bars are 2 cm.

To study safety and functionality of personalized arteries, personal tissue engineered arteries (P-TEA) were transplanted to the carotid artery of five sheep. On two separate sheep, a native carotid artery was isolated, cut in the same way as with the P-TEA transplantation and sutured back as controls for the surgical procedure (sham controls). After reconditioning, the P-TEA had red/pink coloring similar to native tissue (Fig. 2A). Five to seven cm (stretched to approximately nine cm after implantation) of P-TEA was transplanted to the carotid artery with end-to-end anastomosis and metal clips were sutured on the outside of the blood vessel at the anastomosis sites to be able to locate the surgical site during angiography (Fig. 2B). Ultrasound was performed at two weeks and two months, respectively, post-surgery as well as in conjunction with euthanization four months post-surgery. All blood vessels were patent with adequate blood flow at all time points (Fig. 2C). The sheep were anaesthetized, and angiography was performed prior to euthanization. The arteries of all sheep were fully patent with no signs of stenosis or occlusion (Fig. 2D and E).

For one P-TEA, a structure resembling a pseudoaneurysm was found during angiography (data not shown); however, during dissection of the P-TEA it was found that this was a branch on the artery that was ligated during the decellularization/reconditioning process and was filled with blood, thus falsely appearing as a pseudoaneurysm in the angiography. No weakening of the vessel wall was found at this position.

### 3.3 Efficient recellularization of P-TEA *in vivo*

**Fig. 3:**
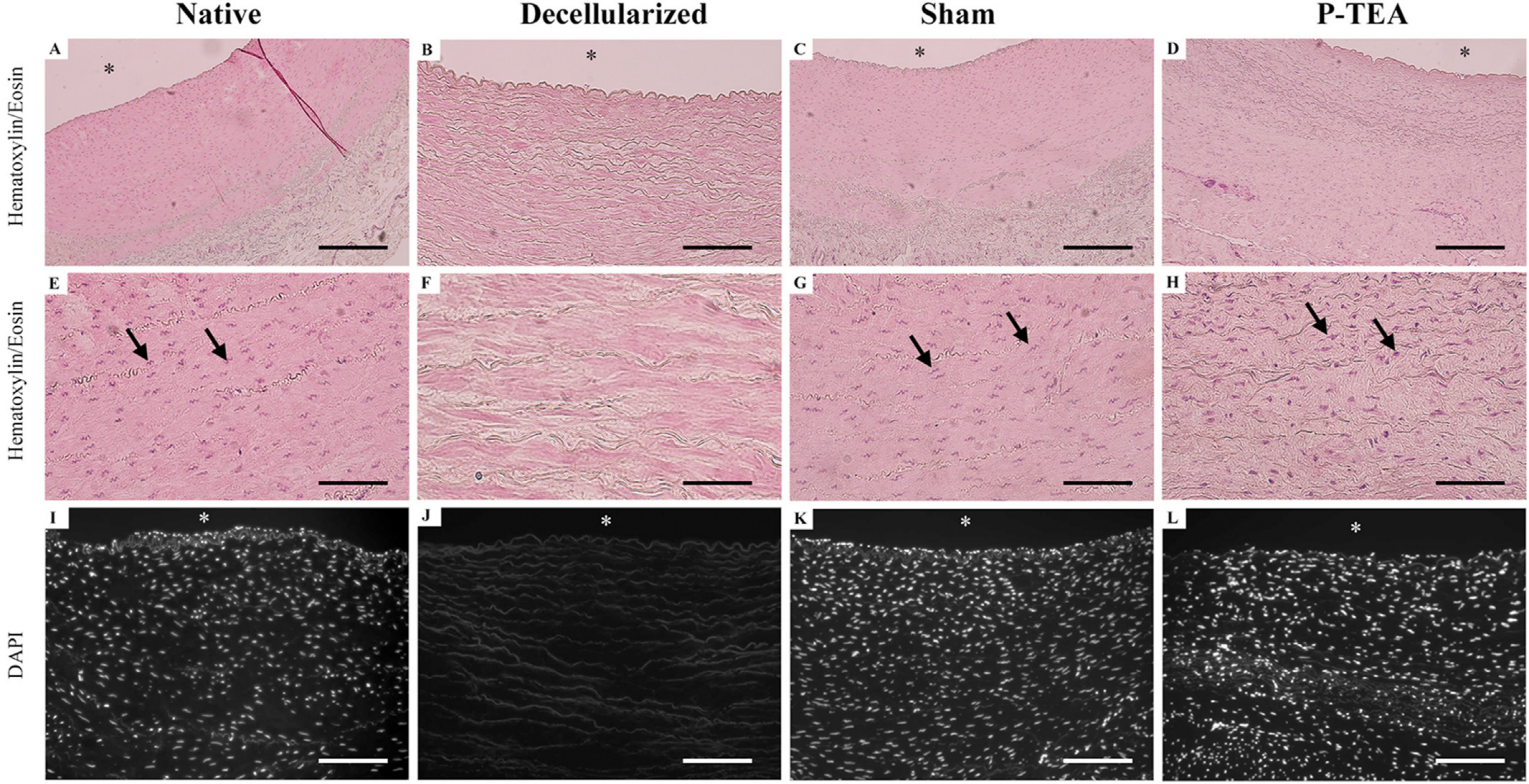
**Hematoxylin/Eosin and 4′,6-diamidino-2-phenylindole (DAPI) stainings of carotid artery after four months *in vivo***. The cells in the native arteries (A, E, I) were efficiently removed in the decellularization process (B, F, J). After four months *in vivo*, sham operated arteries (C, G, K) and the P-TEAs (D, H, L) were comparable cellularized with native artery (A, E, I). Arrows in E, G and H indicate cell nuclei. No nuclei were identified in B, F or J. ∗ = luminal side. Scale bars are 200 μm in A– D and I – L; 50 μm in E − H.

The cellular content of the graft was followed during the P-TEA production and transplantation process using hematoxylin/eosin and DAPI staining (Fig. 3). From the native tissue (Fig. 3A, E and I), there were no cells left after decellularization (Fig. 3B, F and J). After four months *in vivo*, there was equal cellular content and tissue morphology in sham operated arteries (Fig. 3C, G and K) and P-TEA (Fig. 3D, H and L), as compared with the native tissue.

### 3.4 Full luminal endothelialization, and revascularization of the intima

**Fig. 4:**
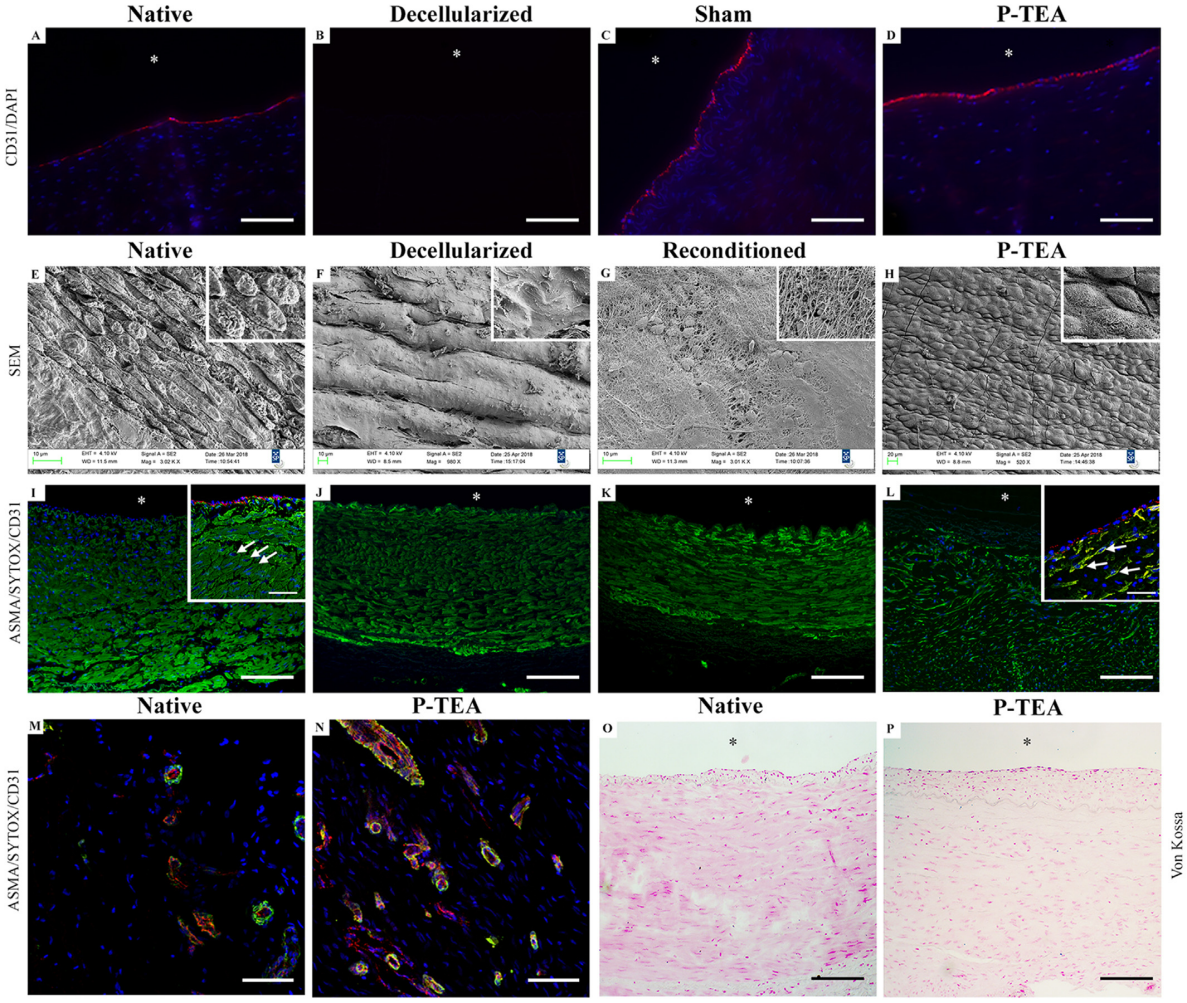
**Characterization of the blood vessel tissue after four months *in vivo*.** The luminal surface was lined with cells expressing the endothelial cell marker CD31 (red), and cell nuclei are indicated with DAPI (blue) (A-D). All cells in the native tissue (A) were removed during the decellularization (B). The sham operated arteries (C) and the personalized tissue engineered arteries (P-TEA) (D) were equivalent with the native tissue four months after transplantation. Scanning electron microscopy shows the endothelial cells on the luminal surface of native artery (E) which were efficiently removed after decellularization (F). Reconditioning added a layer of blood components and cells (G), and the luminal surface was completely recellularized after four months *in vivo* (H)*.* Staining with antibodies against CD31 (red) and alpha smooth muscle actin (αSMA) (green) as well as SYTOX™ Deep Red Nucleic Acid Stain (blue) for nuclei staining during P-TEA production process (I–N). The αSMA expression in native carotid artery cells (I) remained in the tissue after the decellularization (J) and reconditioning (K) process. After four months *in vivo*, the cells in P-TEA showed intracellular expression of αSMA (L) in a remodeled fashion compared with native tissue. Arrows marks cell nuclei and intracellular expression of αSMA. Confocal images of the adventitia in native carotid artery (M) and P-TEA (N) shows capillary revascularization with CD31 expressing cells lined with αSMA-expressing smooth muscle cells and/or pericytes after four months *in vivo.* Von Kossa staining shows no trace of calcification of the P-TEA after four months *in vivo* as comparing native (O) and P-TEA (P). Insets are larger magnifications of the same image. ∗ = luminal side. Scale bars are 200 μm in A – D, I – L and O, P; 150 μm in M, N and 50 μm in inset I and L.

Using staining with antibodies against the endothelial cell marker CD31, we could find that CD31 was equally expressed in the outermost luminal cell layer of native, sham-operated and P-TEA tissue (Fig. 4A–D). To study the morphology of the luminal surface, arterial tissues were analyzed with scanning electron microscopy (SEM). The decellularization process was confirmed to be efficient resulting in a smooth cell free luminal surface comparing native with decellularized artery (Fig. 4E and F). The reconditioning with the animal’s autologous blood establishing a coating with blood components, including few cells (Fig. 4G) and the luminal surface of the P-TEA was well cellularized *in vivo* with a confluent cell layer with endothelial cell morphology lining up in the blood flow direction (Fig. 4H). The morphological differences in the images of native and P-TEA tissue (Fig. 4E and H) are due to fixation artefacts, mostly in the native tissue.

Another important cell type in arteries is smooth muscle cells. Therefore, the expression of the smooth muscle cell marker, alpha smooth muscle actin (αSMA), was mapped during the P-TEA production process and after transplantation with immunohistochemical staining. Even though αSMA is an intracellularly expressed protein, αSMA remained in the artery tissue after decellularization and the expression was intact after reconditioning (Fig. 4I–K). After four months *in vivo*, the cells that had repopulated the P-TEA reorganized the αSMA expression (Fig. 4L) intracellularly (as can be seen in the marked cells in the inset of Fig. 4L) but with a shifted pattern compared with the native tissue (Fig. 4I). Staining with antibodies against CD31 and αSMA (which is also a marker for pericytes in smaller blood vessels) verified capillary revascularization of the arterial adventitia tissue (Fig. 4M and N). Further, Von Kossa staining confirmed that no calcification was present in the transplanted P-TEA after four months *in vivo* (Fig. 4O and P).

### 3.5 Analysis of intimal hyperplasia after P-TEA transplantation

**Fig. 5:**
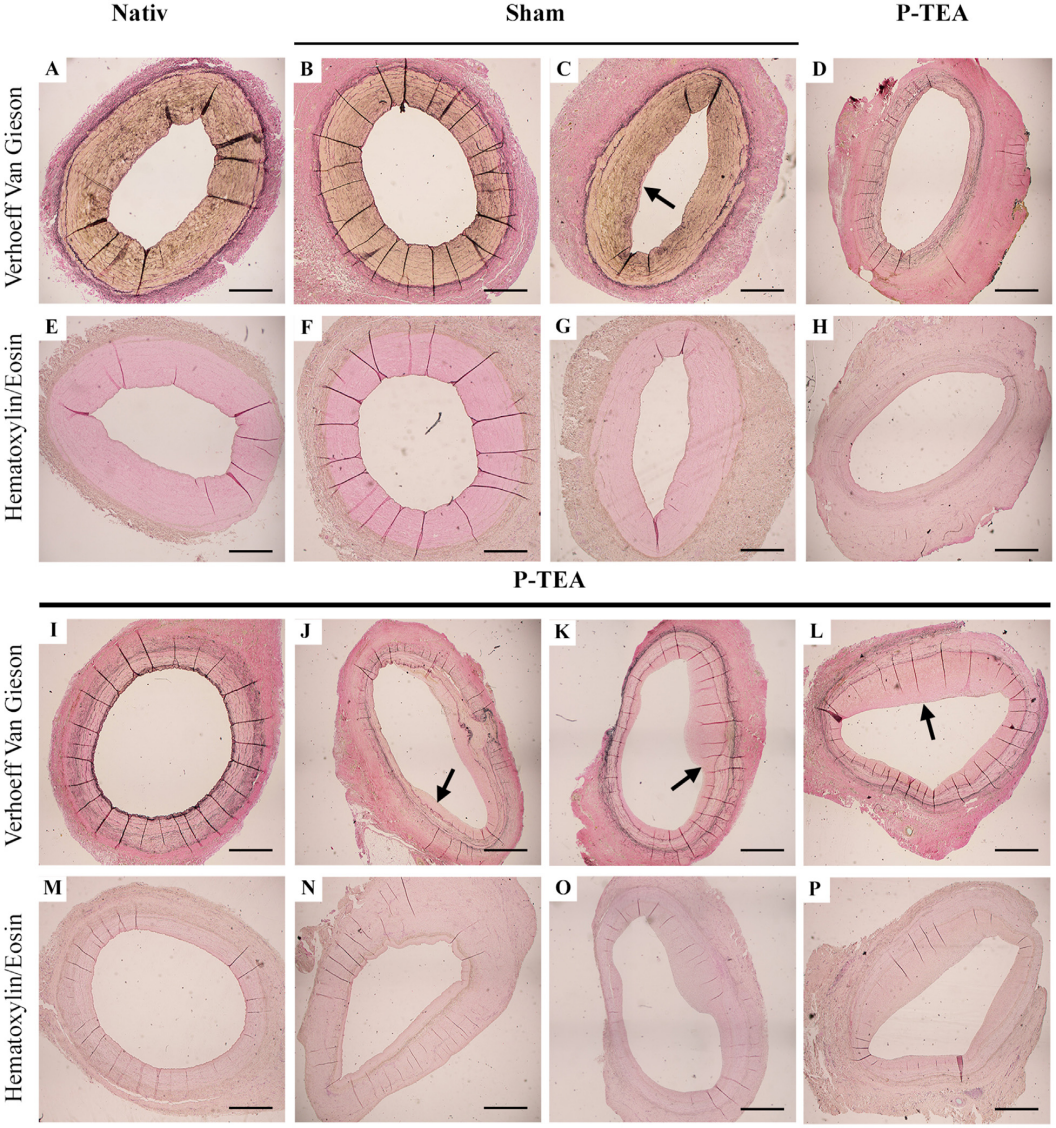
**Characterization of intimal hyperplasia in tissue engineered arteries after four months *in vivo.*** Hematoxylin/eosin and Van Gieson staining’s illustrates the extracellular matrix and intimal hyperplasia (A–P). Native carotid artery without any initial hyperplasia (A, E). Sham operated carotid artery four months after surgery with sections of areas with no (B, F) and small (C, G) intimal hyperplasia. Personalized tissue engineered arteries after four months *in vivo* showing areas with no (D, H, I, M) small (J, N) some (K, O) or more (L, P) intimal hyperplasia. Arrows indicate intimal hyperplasia. Scale bars are 100 μm.

To characterize the extracellular matrix (ECM) structure and formation of intimal hyperplasia after transplantation, native carotid arteries, sham operated arteries and personalized tissue engineered arteries were stained with hematoxylin/eosin and van Gieson stainings (Fig. 5). The native arterial tissue showed no sign of intimal hyperplasia (Fig. 5A and E) whereas sham operated arteries had both vessel segments that were free from intimal hyperplasia (Fig. 5B and F) and some vessel segments that contained a thin layer of intimal hyperplasia (Fig. 5C and G. Intimal hyperplasia is shown as a thickened pink layer of the lumen, indicated by arrow in C). The personalized tissue engineered arteries showed varied areas with no (Fig. 5D, H, I, and M), small (Fig. 5J and N), medium (Fig. 5K and O) and more (Fig. 5L and P) intimal hyperplasia. However, the largest hyperplasia found in the engineered grafts (Fig. 5L and P) was less than 50% of the luminal area inside the internal elastic lamina, which is considerably less than the clinical definition of significant stenosis (<25% of the luminal area open).

To analyze for possible indication of graft rejection, personalized tissue engineered arteries after four months *in vivo* were stained with antibodies against the leukocyte marker CD45 to identify invasion of leukocytes including lymphocytes, monocytes and macrophages (Supplementary Fig. 4). No CD45 positive cells were found in native carotid artery, sham operated artery or personalized tissue engineered transplanted artery, indicating no signs of rejection (Supplementary Fig. 4A–C). A few areas with minor CD45 positive cell clusters were found in the anastomosis region (green in Supplementary Fig. 4D), as expected due to wound healing.

### 3.6 Biomechanical properties

**Fig. 6:**
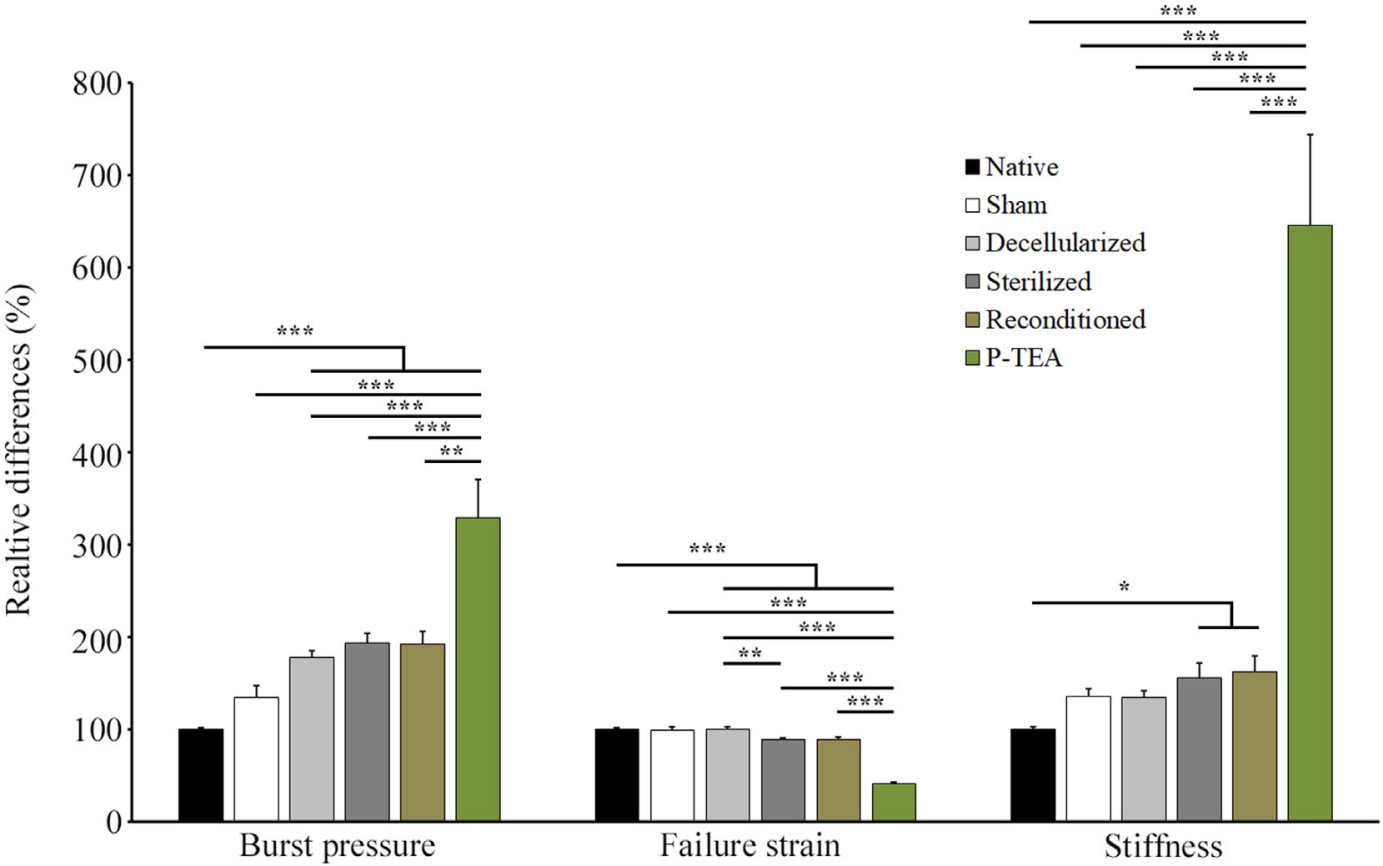
**Biomechanical properties of artery during the tissue engineering process and after four months *in vivo.*** Samples from individual arteries of native (n = 36), sham operated (n = 2), decellularized (n = 36), sterilized (n = 36), reconditioned (n = 36) and after four months *in vivo* (n = 5) were stretched until failure with a biomechanical testing device and analyzed for burst pressure, failure strain and stiffness. ∗ = p < 0.05, ∗∗ = p < 0.01, ∗∗∗ = p < 0.001.

Biomechanical properties of the arteries were assessed over the whole process: in native blood vessels, sham operated, decellularized, sterilized, reconditioned and four months after transplantation. The measurements were performed in a ringlet elongation test. The native arteries showed burst pressure of 1449 ± 83 mmHg, failure strain of 1.08 ± 0.02 and stiffness of 601 ± 53 kPa (average ± SEM). Although not statistically significant, the sham operated arteries had 35% increased burst pressure and stiffness compared with the native arteries. The decellularization, sterilization and reconditioning significantly increased the burst pressure by 77–93% and stiffness by 35–62%, respectively, compared with native arteries, but there was no significant difference in between these groups. In terms of failure strain, the decellularization process had no effect while sterilization decreased the failure strain by 11% which was consistent after reconditioning. After four months *in vivo* the, burst pressure and stiffness were significantly increased while failure strain was significantly decreased, compared with all other groups (Fig. 6).

## 4 Discussion

Tissue engineering, as a part of regenerative medicine, has great potential to cater the patients in need of reconstructive surgery and organ transplantation. As a model for this field, we recently showed that personalized tissue engineered veins could be transplanted to *vena cava* in a porcine model with efficient recellularization and full patency after one month [14]. On the artery side of vascular surgery, there is a great need of small-diameter blood vessels since the available grafts on the market suffer from problems with patency and infections [30,[40], [41], [42]]. Each year, approximately 200,000 CABG and 80,000 bypass for PAD are performed in the USA alone [43], underlining the significance of development of vascular grafts. For arterial grafts to be safe and functional, they need to be non-thrombogenic, show elastic biomechanical properties and provide sufficient burst pressure to withstand arterial pressure of several hundred mm Hg to prevent permanent deformations or aneurysms formation [29]. Another important aspect of transplantation of grafts is to not provoke the immune system and initiate rejection of the graft. To avoid this, our vascular grafts go through a thorough decellularization process (Fig. 1), leaving very small amounts of nucleic acid left in the tissue (Supplementary Fig. 3), well below the recommended level (50 ng DNA/mg dry ECM weight) referred to in the literature [5]. Also, no cells could be identified in the tissue (Figs. 3B, F, J and 4B, J) or on the luminal surface (Fig. 4F) of the graft after decellularization. Several studies have suggested recellularization of decellularized grafts with endothelial cells (ECs) of different sources, such as somatic ECs [6,33], endothelial progenitor cells [16,26,33], induced pluripotent stem cell derived ECs [28] or embryonic stem cell derived ECs [35]. However, recellularization with *in vitro* expanded cells implies disadvantages with risk of inducing genetic variations, mutations, and increased variability of the cells. It is also encumbered with a time-consuming process, high costs, limited availability of cells and regulatory hurdles. Production of vascular grafts should be consistent, operator independent and easily reproducible [13].

In bypass surgery, autologous saphenous vein grafts are often preferable; however, this option is frequently not available because of compromising factors such as varicose or sclerotic vein segments or inadequate vessel diameter. Also, the extended surgical exposure for vascular graft preparation is associated with wound healing problems and infections. The size of the graft is of major importance when it comes to patency of different types of grafts, where the diameter seems to be the major obstacle. Synthetic grafts are functional on large vessels (≥8 mm) but for small-diameter artery prosthetics (≤6 mm), these often have patency problems [10,27,41]. Several publications covering clinical trials with decellularized small to medium diameter grafts including Artegraft®, Solcograft, ProCol®, and SynerGraft® collectively concludes that the outcomes are not satisfactory compared with synthetic conduits [15,[21], [22], [23],^32^,^34^]. Examples of clinical trials performed on a significant number of patients treated, with decellularized grafts with a diameter of 6–8 mm and a length of 10–50 cm, show common complications of aneurysm formation, stenosis and patency issues caused by thrombosis [2,7,19,38,45].

In this study, we evaluated the use of a novel allogenic arterial graft. The length and diameter of the grafts were chosen to fit the sheep carotid animal model, which is in the range of coronary arteries in human [8]. Previous limited performance of decellularized grafts have been suggested to be due to lack of cellularity and exposure of the collagen surface to blood [10,11,32]. In addition to *in vitro* recellularization, discussed above, immobilization of modified peptides to the luminal side of small diameter long bypass arteries for improved *in vivo* endothelialization has been shown efficient for patency in a short-term animal study [24]. With our technique, grafts are decellularized and reconditioned with the recipient’s own blood to facilitate for efficient recellularization *in vivo,* and to prevent thrombosis due to collagen surface exposure. The reconditioning is efficient using only 25 ml of the recipient’s peripheral blood over a seven-day procedure until transplantation (Fig. 1) and results in coverage of the graft surface with a layer of blood components and cells (Fig. 4G). We have previously shown with ROTEM analysis that our reconditioned vascular grafts have better hemocompatibility with reduced activation of the intrinsic coagulation pathway, increased clotting and clot formation time and decreased alpha angle compared with native and decellularized scaffolds [14]. Recellularization of the personalized veins grafts, produced with the same procedure, starts as early as three days after transplantation and is very efficient to repopulate the tissue already two weeks post-surgery [14]. After 4 months *in vivo*, personalized tissue engineered arteries showed recellularization of the deeper layers of the vascular grafts (Fig. 3D, H and L) as well on the luminal side (Fig. 4H). Since the diffusion of oxygen in tissue is only approximately 150 μm [31], revascularization of regenerated tissue is very important. Confocal imaging illustrates that the personalized artery wall was well revascularized 4 months after transplantation (Fig. 4M and N). The cells on the luminal surface, and on the capillaries in the tissue, expressed the endothelial cell marker CD31 as illustrated by immunostaining (Fig. 4A–D, M, and N), indicating that these cells are indeed differentiated endothelial cell. The presence of endothelial cells on the luminal surface is interpreted beneficial for vascular grafts as damage, or absence, of the endothelial cell layer can contribute to thrombosis [1]. Immunostaining against alpha smooth muscle actin (αSMA) revealed presence of smooth muscle cells/pericytes, both in connection with the lumen and in the capillaries of the adventitia of the P-TEA (Fig. 4L, M). Interestingly, αSMA remains in the tissue during decellularization and reconditioning but is reorganized in the recellularized compared with the native tissue (insets in Fig. 4I and L). Histology staining with Von Kossa showed no trace of calcification of the personalized arteries (Fig. 4O and P). In addition, the extracellular matrix of native and graft arterial tissue were comparable on hematoxylin/eosin staining, but the presence of elastin in the graft was differently distributed compared with the native tissue as illustrated with Verhoeff Van Gieson staining (Fig. 5).

Intimal hyperplasia is a common complication of vascular grafts 2–24 months after vascular intervention and causes stenosis in 20–30% of lower limb vein bypasses and 10–30% of coronary graft bypasses [20]. A variety of injuries may be involved in the induction of intimal hyperplasia but endothelial damage is always involved [17]. Most (60–80%) of the intimal hyperplasia is composed of extracellular matrix which is produced from vascular smooth muscle cells (approximately 20%) that have migrated from the media to the intima [17]. In our transplantation study, the sham surgery controls induce some, but very limited, intimal hyperplasia (Fig. 5C and G) while the personalized tissue engineered arteries had local segment spanning from no to pronounced intimal hyperplasia (Fig. 5). However, measuring and comparing the area of the original lumen with the lumen left at the segment with most intimal hyperplasia, the hyperplasia only covered 50% of the lumen which is far from the clinical definition of significant stenosis (<25% of the luminal area open). Importantly, using antibodies against CD45, no infiltrated immune cells were localized within the graft tissue as a sign of tissue rejection, except local clusters at the anastomosis region which we assume are due to healing and tissue reorganization (Supplementary Fig. 4).

For tissue engineered arteries, biomechanical properties are of high importance. They must have mechanical strength, i.e. a burst pressure, sufficient to operate at arterial pressure and secure the vessel from rupture or bursting [29]. Biomechanical evaluation was performed on personalized tissue engineered arteries through the production process as well as after four months *in vivo* and compared with native carotid arteries (Fig. 6). The analysis revealed that the burst pressure increased approximately 2-fold during decellularization while the sterilization and reconditioning had no further effect. Failure strain was not affected by the decellularization, but the sterilization decreased this parameter with approximately 10% which was then consistent during reconditioning. The average stiffness increased somewhat after decellularization to sterilization to reconditioning, but the only groups statistically different were sterilized and reconditioned grafts with approximately 60% increased stiffness compared with native arteries. With only two sham operated arteries, no statistically significant difference was found in biomechanical properties compared with native vessels (Fig. 6). We; however, speculate that the average increase in burst pressure and stiffness of the graft tissue was due to signs of intimal hyperplasia (Fig. 5) and scar tissue around the blood vessel, due to healing after surgery, that was not possible to remove during excision of the specimen. After 4 months *in vivo* the personalized tissue engineered arteries had 3-fold increased burst pressure and 6-fold increased stiffness while failure strain was reduced by 60% compared with native arteries (Fig. 6). The increased burst pressure ensures the personalized arteries to withstand the arterial pressure *in vivo* over time. Our interpretation of the collective data is that the change in distribution of elastin expression and formation of intimal hyperplasia (Fig. 5), together with the scar tissue formation around the vessel as an effect of wound healing after surgery, are the parameters that contributes to the increased stiffness and reduces failure strain. Based on these findings the grafts are safe, no bleeding or aneurisms should be expected. The grafts are stiffer compared with native arteries, but as only minor parts of the arterial system will be replaced by grafts in each application, the effect on the regulatory function of the arterial system is expected to be minor.

A limitation of the study is that a decellularized scaffold was not included as a control for the *in vivo* study. However, the study was designed as a safety study as part of the preclinical development of P-TEA with the aim to assess medium term safety of P-TEA grafts in a large animal model. The study is not intended as a comparative study, and therefore no additional experimental groups were included. The strengths and weaknesses of other grafts such as such as synthetic grafts or commercially available decellularized grafts have been presented previously by others as described above.

In the present study we showed that decellularized arteries, reconditioned with peripheral whole blood, can be safely transplanted into a sheep *in vivo* model. By analyzing transplanted P-TEA four month after surgery we can conclude that the grafts were recellularized to a level equal with the native vein tissue with a protective endothelial cell layer on the luminal graft surface and revascularized adventitia tissue. All P-TEA were fully patent and without signs of coagulation or thrombosis. Intimal hyperplasia was formed with various extent at different locations; however, non were close to significant stenosis, and biomechanical evaluation confirmed that the grafts can stand the arterial blood pressure. These results motivate to proceed with P-TEA in long term studies to further validate the product for clinical studies.

## Author contributions

Conceptualization, Klas Österberg, Lachmi Jenndahl, Yalda Bogestål, Robin Simsa, Tobias Gustafsson-Hedberg, Patrik Stenlund, Sarunas Petronis, Annika Krona, Per Fogelstrand, Raimund Strehl, Joakim Håkansson; Data curation, Klas Österberg, Lachmi Jenndahl, Yalda Bogestål, Robin Simsa, Tobias Gustafsson-Hedberg, Patrik Stenlund, Sarunas Petronis, Annika Krona, Per Fogelstrand, Raimund Strehl, Joakim Håkansson; Formal analysis, Klas Österberg, Lachmi Jenndahl, Yalda Bogestål, Robin Simsa, Tobias Gustafsson-Hedberg, Patrik Stenlund, Sarunas Petronis, Annika Krona, Per Fogelstrand, Raimund Strehl, Joakim Håkansson; Funding acquisition, Joakim Håkansson, Yalda Bogestål, Raimund Strehl; Investigation, Klas Österberg, Lachmi Jenndahl, Yalda Bogestål, Robin Simsa, Tobias Gustafsson-Hedberg, Patrik Stenlund, Sarunas Petronis, Annika Krona, Per Fogelstrand, Raimund Strehl, Joakim Håkansson; Methodology, Klas Österberg, Lachmi Jenndahl, Yalda Bogestål, Robin Simsa, Tobias Gustafsson-Hedberg, Patrik Stenlund, Sarunas Petronis, Annika Krona, Per Fogelstrand, Raimund Strehl, Joakim Håkansson; Project administration, Joakim Håkansson, Yalda Bogestål, Lachmi Jenndahl; Validation, Klas Österberg, Lachmi Jenndahl, Yalda Bogestål, Robin Simsa, Tobias Gustafsson-Hedberg, Patrik Stenlund, Sarunas Petronis, Annika Krona, Per Fogelstrand, Raimund Strehl, Joakim Håkansson; Writing original draft, Klas Österberg, Lachmi Jenndahl, Yalda Bogestål, Robin Simsa, Tobias Gustafsson-Hedberg, Patrik Stenlund, Sarunas Petronis, Annika Krona, Per Fogelstrand, Raimund Strehl, Joakim Håkansson; Writing review and editing. All authors have read and agreed to the published version of the manuscript.

## Ethics statement

The authors confirm that the ethical policies of the journal, as noted on the journal’s author guidelines page, have been adhered to and the appropriate ethical review committee approval has been received.

## Data availability

The data that support the findings of this study are available from the corresponding author upon reasonable request.

## Declaration of competing interest

Lachmi Jenndahl, Tobias Gustafsson-Hedberg, Robin Simsa and Raimund Strehl were employees of the company VERIGRAFT AB which contributed financially, including salaries and study costs, and by providing laboratory space.
