# Supplementary material for: Personalized tissue-engineered arteries as vascular graft transplants: A safety study in sheep
Source: Regen Ther. 2022 Sep 7;21:331–41. doi: 10.1016/j.reth.2022.08.005 (PMC9463533; doi:10.1016/j.reth.2022.08.005)
Supplement: Multimedia component 1 [file mmc1.docx]

**Supplementary Material**

| A | | | | | | | | | | | | B | | | | | | | | | | | | C | | | | | | | | | | | |
| --- | --- | --- | --- | --- | --- | --- | --- | --- | --- | --- | --- | --- | --- | --- | --- | --- | --- | --- | --- | --- | --- | --- | --- | --- | --- | --- | --- | --- | --- | --- | --- | --- | --- | --- | --- |
| Native | | | DC | | | Sterile | | | RC | | | Native | | | DC | | | Sterile | | | RC | | | Native | | | DC | | | Sterile | | | RC | | |
| 1 | 2 | 3 | 1 | 2 | 3 | 1 | 2 | 3 | 1 | 2 | 3 | 1 | 2 | 3 | 1 | 2 | 3 | 1 | 2 | 3 | 1 | 2 | 3 | 1 | 2 | 3 | 1 | 2 | 3 | 1 | 2 | 3 | 1 | 2 | 3 |

**Supplementary Figure 1. Schematic overview of sample preparation for biomechanical testing.**

For measurement of native, decellularized and reconditioned arteries, samples were taken within a single vessel to avoid biological variability between donors. Each artery was divided in three sections (A-C) for which three ring segments were cut off and selected for testing. DC = Decellularization, RC = Reconditioning. Measurements on P-TEA and sham operated vessels four months after surgery were performed directly after explantation.

**Supplementary Table 1.** Summarized data of the individual blood vessels transplanted in the current study on arteries transplanted in sheep and in our previous study on veins transplanted in pig (Håkansson *et al.* 2021). For the graft length of arteries, two numbers are noted: The one without parenthesis is the length before implantation, the one inside parenthesis is when sutured in place. Some tension was added to the graft to avoid folding.

| **Current artery graft study in sheep** | | | |
| --- | --- | --- | --- |
| **Sheep Nr** | **Time *in vivo* (days)** | **Graft length (mm)** | **Luminal diameter (mm)** |
| 1 | 110 | 65 (90) | 3.8 |
| 2 | 110 | 65 (90) | 3.5 |
| 3 | 111 | 65 (90) | 3.3 |
| 4 | 111 | 70 (90) | 3.9 |
| 5 | 126 | 50 (70) | 3.5 |
| **Previous vein graft study in pig (Håkansson *et al.* 2021)** | | | |
| **Pig Nr** | **Time *in vivo* (days)** | **Graft length at implantation (mm)** | **Luminal diameter at implantation (mm)** |
| 1 | 3 | 30 | 11 |
| 2 | 15 | 20 | 12 |
| 3 | 17 | 30 | 14 |
| 4 | 29 | 37 | 14 |
| 5 | 29 | 32 | 13 |
| 6 | 37 | 40 | 14 |

**
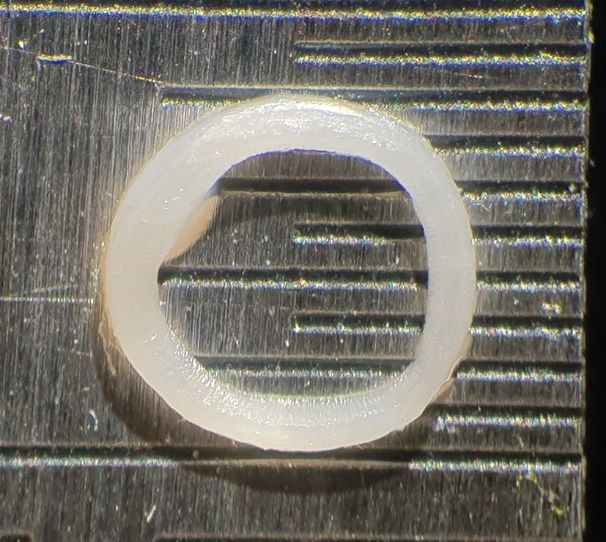
**

**Supplementary Figure 2 Carotid sheep graft.** Carotid sheep graft on a mm scale where the smallest lines represent 0.5 mm.


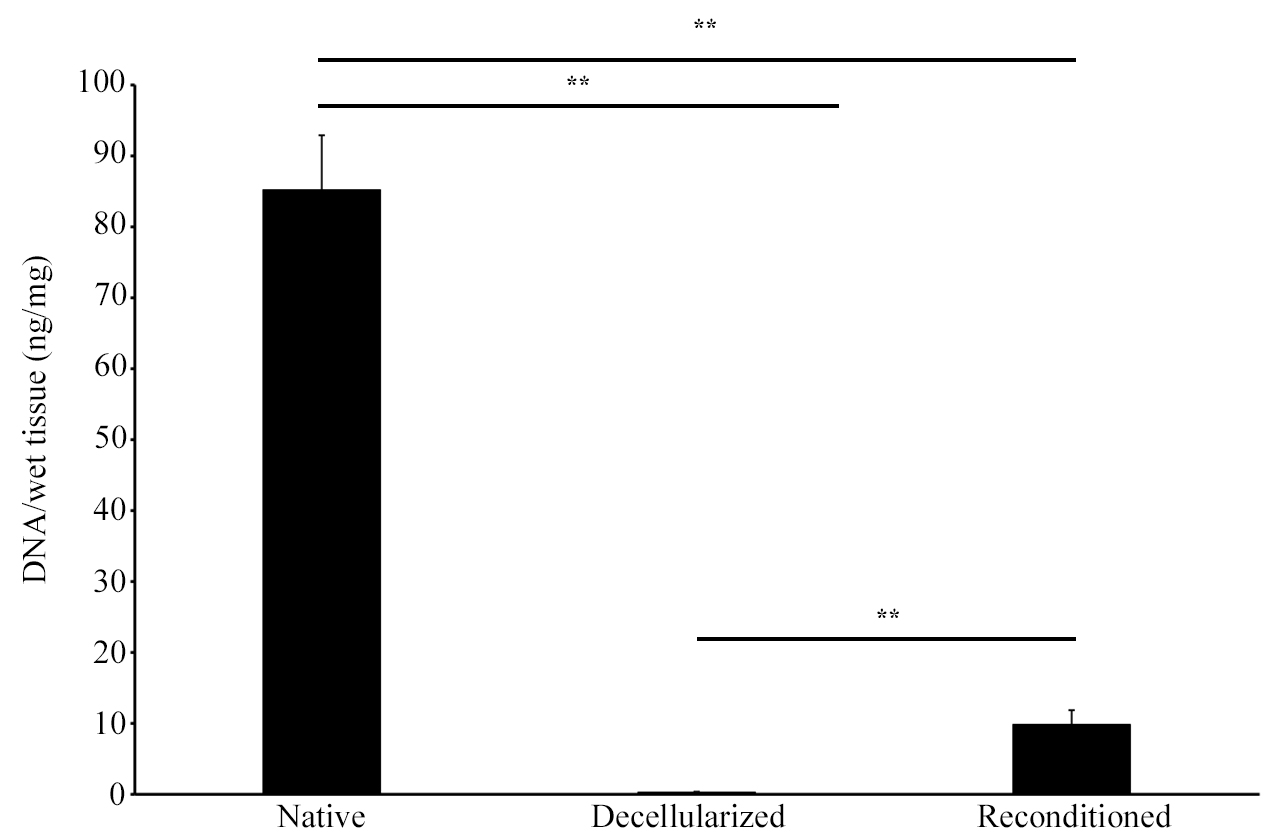


**Supplementary Figure 3 DNA content during preparation of personalized tissue engineered artery.**

DNA content in native, decellularized and reconditioned vascular grafts. Staples represents average and error bars SEM. ** = p<0.01, (n=6).


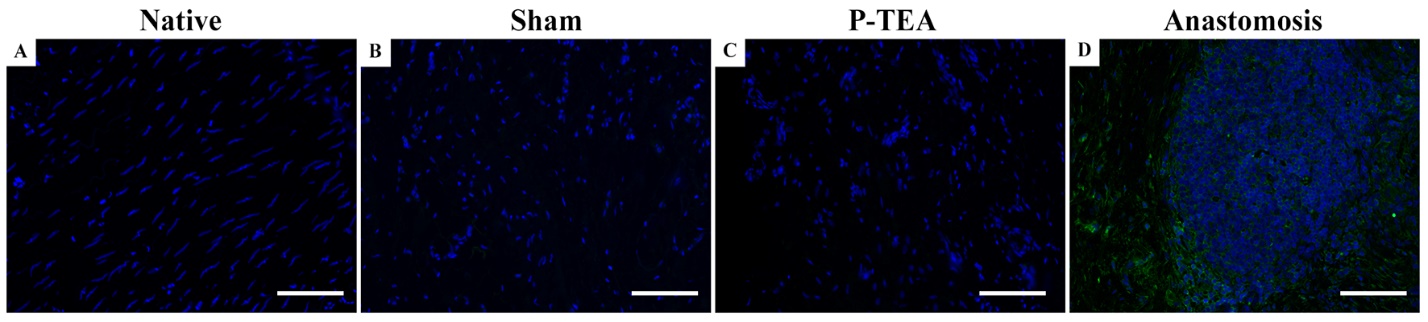


**Supplementary Figure 4 Identifying immune cells in the arteries.** Immunostaining with antibodies against the leucocyte marker CD45 in native (A), sham operated artery (B), personalized tissue engineered artery (C) and the anastomosis site between the native and graft tissue (D). Blue indicate DAPI (nuclei) and green CD45. Scale bars are 200 µm.
